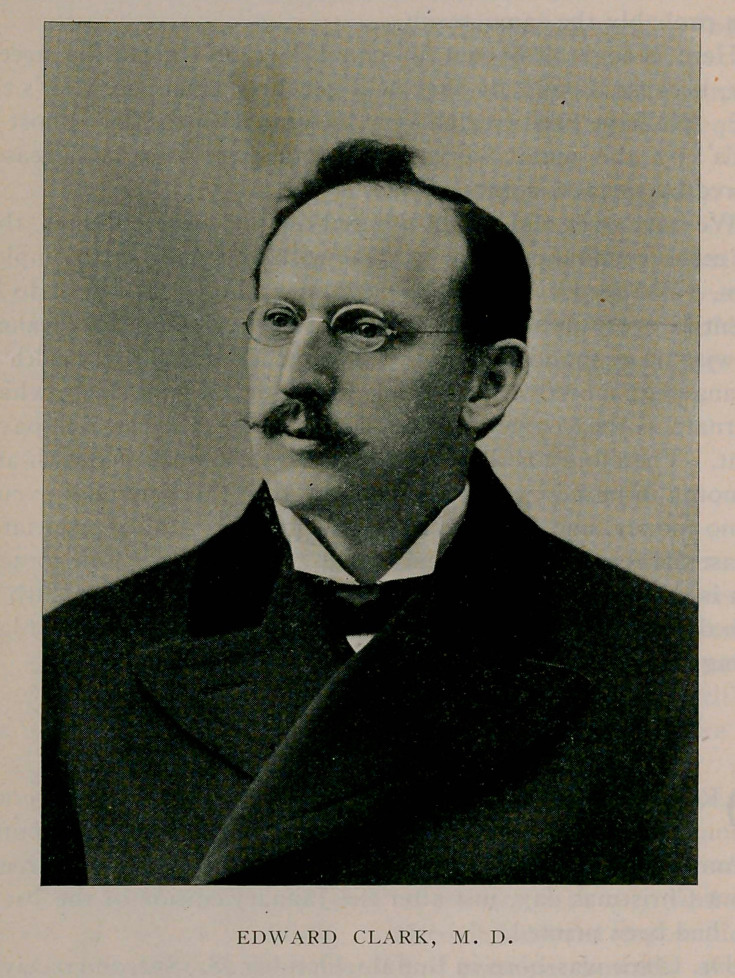# The Deputy Commissioner of Health

**Published:** 1902-02

**Authors:** 


### The Deputy Commissioner of Health.

DR. EDWARD CLARK has been appointed deputy commissioner of health, vice Walter D. Greene promoted commissioner. The appointment was announced by Dr. Greene about Christmas day, just after the January edition of the JOURNAL had been printed.

Dr. Clark was born in Buffalo, October 28, 1852, and received his preliminary education in the public schools of this city, including two years at the high school. He became a teacher, himself, and through this means earned money to prosecute his medical studies. He entered the Cincinnati College of Medicine and Surgery in 1876, and received his doctorate degree from the medical department of the University of Buffalo, February 25,

1880. He immediately began the practice of medicine in his native city which he has continued to the present time.

During these years he has held the office of postmortem examiner to the coroner, physician to the county jail, sanitary inspector for the health department, and, finally, health physician, as it was then termed, which latter office he held in 1888.

EDWARD CLARK, M. D.

During his term there was an outbreak of smallpox, that he stamped out in short order. His experience then has served the city a good turn in the present epidemic.

Dr. Clark taught anatomy at the Niagara University for some years, and was a lecturer on special surgery at the University of Buffalo for about two years. He is attending surgeon at the

Erie County Hospital and was surgeon of the Emergency Hospital in 1885, and assistant at the Sisters' of Charity Hospital at the same time. He is a member of the Medical Society of the County of Erie, of which he is also treasurer; a member of the Medical Society of the State of New York, and of the American Public Health Association. He is the author of several medical monographs. He is a member of the legislative committee of the Medical Society of the County of Erie, and has attained reputation as a fearless, clear, and forceful speaker before legislative committees at Albany.

From all this it would appear that not only is Dr. Clark what we like to term in this country a self-made man, but his training and experience has been such as to particularly fit him for service as one of the chief officers of the health department, being second in authority and, during the absence of the commissioner becomes the temporary head. The commissioner, the city, and the profession of medicine, one and all, are fortunate in the appointment of so energetic, capable, fearless, and honest a deputy commissioner of health.

---

The *Buffalo Morning Express* said editorially December 25, 1901, of Dr. Clark's appointment:

A majority of physicians would agree that Dr. Edward Clark is the man best qualified to be assistant health officer. He was health physician under the old charter and he has been connected with the department almost continuously ever since. He has held various other offices of a similar character. Apropos of the present situation, he has had great experience in caring for smallpox. He is a man of proved executive ability and energy. He is both a student and a skilful practitioner of medicine.

Even those physicians who objected to Dr. Greene's promotion have only words of praise for his new chief assistant. Under Drs. Greene and Clark the health department should not decline in efficiency or public esteem.

---

Dr. Ernest Wende on his retirement from the office of commissioner of health of the City of Buffalo was the recipient of a public dinner, tendered by the citizens independent of political or professional affiliation.

The invitation to Dr. Wende read as follows:

Buffalo, December 27, 1901.

*Dr. Ernest Wende, No. 471 Delaware Avenue, Buffalo:*

*My Dear Doctor Wende.*—As an expression of their appreciation of your efforts as health commissioner of the City of Buffalo in safe guarding the public health, in advancing the knowledge of the public in sanitary science and in maintaining the efficiency of your department to an extent that has won for you and it a national reputation, the undersigned, with many other citizens, desire to tender you a banquet, to be held on the evening of January 4, 1902, at 7 o'clock, at the Ellicott Club.

Will you kindly indicate at your earliest convenience your desire in this matter?

| | | |
|---|---|---|
| Charles Cary, | J. J. Albright, | Charles G. Stockton, |
| John H. Williams, | F. P. Lewis, | John G. Milburn, |
| John H. Pryor, | William A. Douglas, | H. M. Watson, |
| J. N. Larned, | Charles E. Walbridge, | S. M. Clement, |
| L. L. Babcock, | John Parmenter, | Edmund Hayes, |
| J. N. Adam, | Wm. B. Wright, Jr., | Simon Fleischman, |
| E. R. Rice, | Ottomar Reinicke, | Adelbert Moot. |

Here is Dr. Wende's reply:

*Dr. Charles Cary, J. J. Albright, S. M. Clement and others:*

*Gentlemen*—I know not why a banquet should be tendered me as an expression of appreciation for my efforts as health commissioner of the City of Buffalo.

The conscientious performance of the labors connected with that responsible office was simply an act of duty and does not entitle me to any extraordinary manifestation of public approval. Nevertheless, it is a great satisfaction to receive an expression of approbation, after ten years' service, from those whose esteem I have endeavored to deserve, and with grateful thanks I yield to your desire by accepting the date you name, January 4, 1902, for the proposed dinner.

Yours most respectfully,

ERNEST WENDE.

BUFFALO, December 28, 1901.

At the appointed time about 170 diners assembled at the club rooms to welcome the guest of honor. The material feast was all that could be desired and after it was over speeches were made approving Dr. Wende's administration, and deprecating his retirement. Dr. Charles Cary presided and the speakers were John G. Milburn, J. N. Larned, John H. Pryor, and Henry R. Hopkins.

Dr. Charles G. Stockton offered resolutions commending Dr. Wende's administration of the health department, and Mr. George P. Sawyer in behalf of those present presented to Dr. Wende a magnificent silver cup. In making the presentation Mr. Sawyer paid deserved tribute to the ability and faithfulness of its recipient.

In reply Dr. Wende said:

I deeply appreciate the kind words spoken of my administration of the health department, and the kind words accompanying the beautiful cup which you have given to me. They exceed any merit of my own, yet I accept them with pride and gratitude as evidence of your regard and approval of my official conduct. I recognise in your compliments appreciation of that honorable professional ambition, which should characterise the work of any official. Sanitary science is based on correct interpretation of facts, and if any benefits have followed my efforts it was because of my conscientious administration of a public trust. I have tried to educate the people that they must take care of their health, if they would live. Your keepsake, this beautiful cup, shall have an honored place among my treasures, and I shall always remember this occasion with unalloyed pleasure. I regret that I cannot more adequately express the gratitude I feel toward you.

The employees of the health department presented to Dr. Wende, as he retired from the office of health commissioner, a solid silver table service of more than 100 pieces in a chest of silk-lined oak. Herbert W. Hill, the city chemist, made the presentation speech and Dr. Wende made appropriate response.

---

THE Maltine Company, of Brooklyn, N.Y., has quite surprised the medical profession, if not astonished it, by the offer of two prizes—one for $1,000, and one for $500—for essays upon preventive medicine. In our regular advertising pages will be found an announcement of this action, and in its appropriate place under "Literary Notes" will be found a full statement of the conditions governing the award.

Furthermore, in our correspondence column Mr. Charles C. Heuman, the secretary of the company, gives an elaboration of the scheme that deserves to be read in its relation to the other statements referred to. This was a personal letter to the editor, but it states the object so well and is such an appropriate supplement to the others, that we prefer to let the accomplished secretary, himself, speak for the Company, rather than to attempt such a rôle ourselves, which we feel sure would be less graceful and effective.

This competition will, of course, be eagerly entered by hundreds of capable physicians, and the result will be looked forward to with anxious expectation. It will undoubtedly yield valuable contributions to the literature of preventive medicine. The Maltine Company deserve congratulations upon its liberal spirit and generous offer.

THE anti-spitting ordinance is to be enforced as it applies to the street cars. A recent conference between the health commissioner, Dr. Greene, and the superintendent of the Buffalo Street Railway Company was held on the subject, which resulted in posting the following notice in the cars:

> Spitting on the floor of this car is prohibited by law and is punishable by a fine of from $2.00 to $100 for each offense. Policemen are instructed to arrest and remove from the car any passenger violating the provisions of this act.
>
> WALTER D. GREENE, M. D., Health Commissioner.

Would it not be wise to appoint each conductor a special officer to aid in the enforcement of the law?

---

THE Commissioner of Pensions has reduced the estimates for his bureau for the next fiscal year by more than $5,000,000 less than for the current year. This is a wholesome showing and is in accordance with the predictions of the commissioner in previous reports. The economic, patriotic and able administration of the pension office by Commissioner Evans, ought to be the pride of every American citizen. Such officials should be kept in public office as long as they can be persuaded to stay.

---

DISTRICT-ATTORNEY JEROME, of New York, has set a splendid example of official duty in his action regarding the recent tunnel horror at New York. It is related that as soon as he heard of it, sitting in his office and busily engaged though he was, he dropped everything and proceeded to the scene, thus familiarising himself with the facts, before they could be changed or colored by lapse of time. He also appeared at the coroner's court and conducted the case for the county, successfully opposing the appearance of counsel for any accused person or corporation. When an attorney for the people manifests this high conception of his duty, and sets up these high ideals, the other similar officers throughout the state may well ask whether they had not better bestir themselves on similar lines.

---

THE osteopathy bill is up again in the legislature. This subject is so well understood through its full discussion in the newspapers and medical journals last year and this, that very little remains to be said about it. Recent editorials in the *Buffalo Enquirer* and *New York Tribune* have presented the question with calmness and intelligence. We advise our members of the legislature to read them. The *Enquirer's* date is January 22, and the *Tribune's* January 23. It would seem to be the height of inconsistency, if not folly, for the legislature to set up a standard for medical practice that is nearly perfect, and then proceed to nullify it by class legislation.

## Figures and Tables

**Figure f1:**